# Mobilizing diversity: transposable element insertions in genetic variation and disease

**DOI:** 10.1186/1759-8753-1-21

**Published:** 2010-09-02

**Authors:** Kathryn A O'Donnell, Kathleen H Burns

**Affiliations:** 1 Department of Molecular Biology and Genetics, The Johns Hopkins University School of Medicine, Baltimore, MD, USA; 2 The High Throughput Biology Center, The Johns Hopkins University School of Medicine, Baltimore, MD, USA; 3 Department of Pathology, The Johns Hopkins University School of Medicine, Baltimore, MD, USA; 4 Department of Oncology, The Johns Hopkins University School of Medicine, Baltimore, MD, USA

## Abstract

Transposable elements (TEs) comprise a large fraction of mammalian genomes. A number of these elements are actively jumping in our genomes today. As a consequence, these insertions provide a source of genetic variation and, in rare cases, these events cause mutations that lead to disease. Yet, the extent to which these elements impact their host genomes is not completely understood. This review will summarize our current understanding of the mechanisms underlying transposon regulation and the contribution of TE insertions to genetic diversity in the germline and in somatic cells. Finally, traditional methods and emerging technologies for identifying transposon insertions will be considered.

## Introduction

In the 60 years since Barbara McClintock first discovered transposable elements (TEs), it has become increasingly recognized that these mobile sequences are important components of mammalian genomes and not merely 'junk DNA'. We now appreciate that these elements modify gene structure and alter gene expression. Through their mobilization, transposons reshuffle sequences, promote ectopic rearrangements and create novel genes. In rare cases, TE insertions which cause mutations and lead to diseases both in humans and in mice have also been documented. However, we are at the very earliest stages of understanding how mobile element insertions influence specific phenotypes and to the extent to which they contribute to genetic diversity and human disease.

TEs are categorized into two major classes based on their distinct mechanisms of transposition. DNA transposons, referred to as Class II elements, mobilize by a 'cut-and-paste' mechanism in which the transposon is excised from a donor site before inserting into a new genomic location. These elements are relatively inactive in mammals, although one notable exception is a piggyBac element recently identified to be active in bats ([1], R Mitra and N Craig, personal communication). In humans, DNA transposons represent a small fraction (3%) of the genome [2]. Retrotransposons, also known as Class I elements, mobilize by a 'copy-and-paste' mechanism of transposition in which RNA intermediates are reverse transcribed and inserted into new genomic locations. These include long terminal repeat (LTR) elements such as endogenous retroviruses, and non-LTR retrotransposons. Endogenous retroviruses are remnants of viruses that have lost the ability to re-infect cells. These elements, which comprise 8% of the human genome, perform reverse transcription in cytoplasmic virus-like particles [2]. In contrast, non-LTR retrotransposons undergo a distinct mechanism of transposition whereby their RNA copies undergo reverse transcription and integration through a coupled process that occurs on target genomic DNA in the nucleus [3-5].

Of all mobile element families, only the retrotransposons remain actively mobile in the human and primate genomes and serve as ongoing sources of genetic variation by generating new transposon insertions. LINEs (long interspersed nucleotide elements) represent the most abundant autonomous retrotransposons in humans, accounting for approximately 18% of human DNA. Non-autonomous elements such as SINEs (short interspersed nucleotide elements) and SVAs [hybrid SINE-R-VNTR (variable number of tandem repeat)-*Alu *elements] require LINE-1 (L1) encoded proteins for their mobilization [2,6-9]. Together, SINEs and SVA elements occupy ~13% of the human genome.

It is both impressive and puzzling that almost half of our genome is composed of these repeat sequences. Evolutionary paradigms dictate that useless elements and harmful TE insertions events should be selected against, while beneficial insertions should gain a selective advantage and thus be retained. Indeed, the most successful transposons have co-evolved with their hosts. Most transposable element insertions are expected to have few consequences for the host genome and, therefore, have little to no impact on gene function [10]. Rarely, transposon insertions will have a deleterious effect on their host genome, resulting in human disease. To date, approximately 65 disease-causing TE insertions (due to L1, SVA and *Alus*) have been documented in humans [11]. Less frequently recognized are instances in which transposons have made innovative contributions to the human genome. In these cases, mobile element sequences have been co-opted by the host genome for a new purpose. For example, approximately 150 human genes have been derived from mobile genetic sequences [2,12,13]. Perhaps the best studied example of a domesticated transposon is the RAG1 endonuclease, which initiates V(D)J recombination leading to the combinatorial generation of antigen receptor genes. The RAG endonucleases have been demonstrated to function as transposases *in vitro*, providing strong support for the idea that the V(D)J recombination machinery evolved from transposable elements [14-16].

In this review, we examine mechanisms of transposon regulation and discuss how TE insertions account for genetic diversity in the germline and in somatic cells. Traditional methods and recently developed technologies for identifying these insertions will also be considered.

## Mechanisms of TE regulation

Expansion of mobile elements occurs when *de novo *insertions are transmitted through the germline to subsequent generations. Indeed, successful metazoan transposons often show germline-restricted expression. As TEs pose a significant threat to genome integrity, uncontrolled activation of these elements would imperil both the host and the element. It appears that, as a consequence, metazoan genomes have evolved sophisticated mechanisms to limit the mobilization of these elements.

DNA methylation is, perhaps, the most well understood mechanism involved in the regulation of TEs in the germline of plants, fungi and mammals [17-20]. Cytosine methylation silences LTR and non-LTR elements by blocking transcription of retrotransposon RNA. Host suppression mechanisms appear to function post-transcriptionally as well. For example, the premature termination of transcription and alternative splicing inhibits expression of LINE-1 elements [21,22]. A family of RNA/DNA editing enzymes with cytosine deaminase activity known as APOBECs (apolipoprotein B mRNA editing enzyme, catalytic polypeptide) has been found to inhibit LINE-1, *Alu*, and mouse IAP (intracisternal A particle) elements [23]. Interestingly, suppression of retrotransposons by APOBECs does not require any editing activity, suggesting that these proteins may carry out a novel function in addition to their ability to act as cytosine deaminases. Several groups have proposed that APOBECs may sequester retrotransposon RNA in cytoplasmic complexes, although additional studies are warranted in order to prove this hypothesis [24,25]. RNA interference is believed to control retrotransposition [26], although the observed effect in mammalian cells *in vitro *is modest [27,28].

Recently, a novel form of mobile element control has emerged that involves small RNAs in germ cells [29]. At the heart of this pathway is a class of small RNAs [piwi-interacting RNA (piRNAs)] that bind to the germline-restricted Piwi subclass of the Argonaute family of RNA interference effector proteins. In *Drosophila*, piRNAs are enriched in sequences containing retrotransposons and other repetitive elements. Disruption of Piwi proteins results in the reduction in piRNA abundance and transposon derepression [30,31]. A series of elegant studies in *Drosophila *and zebrafish directly implicated Piwi proteins in piRNA biogenesis to maintain transposon silencing in the germline genome [32-34]. These findings have led to the idea that piRNAs might immunize the *Drosophila *germline against potentially sterilizing transposition events [32,35].

Mutations in two mouse Piwi orthologues (*Mili *and *Miwi2*) result in the loss of TE methylation in the testes, transposon derepression and meiotic arrest during spermatogenesis [36,37]. Interestingly, the mouse MAELSTROM (MAEL) protein was found to interact with MILI and MIWI in the germline-specific structure *nuage *[38], suggesting that MAEL may also function in this pathway. Nuage (French for 'cloud') is a perinuclear electron-dense structure found in the germ cells of many species [39]. In flies, Mael is required for the accumulation of repeat-associated small interfering RNAs (siRNAs) and repression of TEs [40]. Soper *et al*. demonstrated that loss of *Mael *leads to germ cell degeneration (at the same point in meiosis as *Mili *and *Miwi2 *mutants) and male sterility in mice [41]. In addition, they provided evidence that the mammalian MAEL protein is essential for the silencing of retrotransposons and determined that early meiosis is a critical timepoint when transposon control is established in the male germline. More recently, a similar role for another germ cell protein, GASZ, has been uncovered [42]. Given that MAEL, MILI, MIWI and GASZ all localize to *nuage *(chromatoid body in mammals), this structure is likely where the piRNA pathway defends the germline genome from the invasion of unchecked transposable elements.

## Consequences of TE insertions in the germline

New retrotransposon insertions arising in or passing through the germline can lead to constitutional genetic diseases in humans, although these are uncommonly recognized events. Not surprisingly, it is the TE families most actively propagating themselves in the human genome that are found to cause these diseases, namely and in order of prevalence, *Alu*s, L1 s and SVAs.

As a result of male hemizogosity for the X chromosome, loss-of-function mutations affecting boys have been disproportionately described. Examples include numerous *Alu *and L1-induced coagulopathies by disruption of coagulation factor VIII or factor IX [43,44], *Alu *and SVA insertions causing immunodeficiency by disrupting BTK [45] and LINE-1 insertions in the large dystrophin locus resulting in muscular dystrophies and cardiomyopathies [46-48].

Autosomal transposon insertions leading to human disease have also been described. These tend to phenocopy otherwise autosomal dominant diseases caused by mutation of the transposon target locus. Examples include an intronic *Alu *insertion disrupting function of the NF1 tumour suppressor and causing clinical neurofibromatosis [49] and a small number of independent *Alu *insertions affecting fibroblast growth factor receptor 2 (FGFR2) and causing malformations with craniosynostosis categorized as Apert syndrome [50,51].

Thus, while most *de novo *insertions are likely to be passed on as phenotypically silent repeats, it is well established that transposon insertions are relevant to human clinical genetics and can have severe phenotypic consequences in rare cases [52,53]. There remains significant speculation about whether our understanding of this is limited by the technical difficulties in detecting these sequences (discussed below) or if retrotransposition is indeed effectively prevented so that *de novo *insertions uncommonly underlie human disease.

## Transposon insertions in somatic cells

There is a widely accepted belief that truly 'selfish' genetic elements must mobilize selectively in the germline or during early development in order to guarantee their evolutionary success. However, recent evidence from several laboratories challenges this notion. Belancio and colleagues reported that both full-length and processed L1 transcripts are detected in human somatic tissues as well as in transformed cells [54]. Kubo and colleagues demonstrated that L1 retrotransposition occurs in a low percentage of primary fibroblasts and hepatocyes when an adenoviral delivery system is employed to express the L1 element [55]. Additionally, L1 somatic retrotransposition events have been discovered in blastocysts from transgenic mouse and rat models expressing a human L1 element [56]. These data suggest that L1 elements contribute to somatic mosaicism. The proposed model is that L1 RNA transcribed in germ cells is carried over through fertilization and then integrates during embryogenesis. At least one case of human disease appears traceable to a similarly timed insertion in a mosaic mother who transmitted the insertion to her child [57]. Somatic insertions have also been identified in mouse models expressing a synthetic mouse L1 element [58]. However, in these studies the elements are expressed from heterologous promoters.

Gage and colleagues reported that L1 retrotransposition occurs in cultured mouse neuronal progenitor cells and in a mouse model harboring a human L1 element [59]. Based on these findings, it is hypothesized that L1 retrotransposition events might contribute to neuronal plasticity and, perhaps, individuality. In a recent follow-up study, Gage and colleagues detected an increase in the copy number of endogenous L1 in several regions of the adult human brain compared to the copy number of these elements in liver or heart genomic DNA from the same person [60]. In some cases, the brain samples contained ~80 additional copies of L1 sequence per cell. The functional consequences of these findings are, as yet, unknown and many questions remain regarding whether these brain-specific L1 insertions could potentially affect neuronal cell function. Despite these unanswered questions, interesting parallels can be drawn between neuronal cell diversity and the immune system. Namely, immune cells are the only other somatic cell type known to undergo an orchestrated genomic sequence-level alteration process whereby genes that encode antibodies are shuffled to create a host of antibodies that recognize a large number of antigens. Given that the human nervous system embodies a seemingly equally astounding degree of complexity and variability, it is possible that L1 mobilization may play a role in somatic cell diversity. Yet, dysregulation of transposon control mechanisms in the brain might also contribute to neurologic disease.

The extent to which TE insertions may generate diversity in somatic cells remains largely unexplored. It remains unclear why transposons do not hop more often in somatic cells. One possibility is that a transposon defense pathway present in somatic cells has yet to be discovered. One potential candidate involved in somatic TE repression might be the P body (processing body), the somatic equivalent of the germline-specific structure *nuage*. These cytoplasmic structures contain enzymes involved in RNA turnover, including members of the RNA-induced silencing complex. L1 RNA and ORF1 have been shown to accumulate in stress granules, which associate with P bodies in somatic cells [61]. It is tempting to speculate that these structures somehow coordinate repression of TEs in somatic cells, although additional studies are necessary.

## Mobile elements and cancer

A hallmark of neoplastic proliferation is the accumulation of somatic genetic changes. Many types of cancer involve recurrent karyotypic abnormalities or other forms of genomic instability. The roles that mobile elements may play in these processes have been largely speculative. In humans, constitutionally integrated transposons have fairly well established roles as substrates in non-allelic homologous recombinations; but do they also potentiate oncogenesis by somatic expression of, for example, genotoxic L1-encoded proteins? Beyond this, are they capable of completing retrotransposition in such a way as to inactivate key tumor suppressor genes? In rare cases, they do appear to do the latter. For example, LINE-1 retransposition was shown to be an important step in the development of a colon cancer when a tumor-specific exonic insertion in adenomatosis polyposis coli (*APC*) was described [62]. Using an approach that combines linker-mediated polymerase chain reaction (PCR) and high-throughput sequencing (to be discussed in the next section), Iskow and colleagues recently identified several L1 insertions in human lung tumor samples [63]. Although mutations with functional consequences were not demonstrated, these data support a model whereby L1 activity creates tumor genomic heterogeneity. This underscores at least possible roles for transposon insertions in tumor progression.

Suggesting that transposons may have tumor-specific effects dependent on their expression is the observation that demethylation of their promoter sequences have been described in several human tumors. Several examples for the L1 promoter are described in Table 1. In most cases, studies have not convincingly carried these observations further to document that this results in full-length LINE-1 transcripts or expression of functional ORF1p and ORF2p proteins. In a few documented cases, full length L1 RNA in cancer cell lines [54,64] and expression of ORF1p in pediatric germ cell tumors [65] and breast cancer [66] have been shown. Thus, it is possible that tumors provide an environment where transposition events can occur and be selected for in transformation. In at least one animal model, the mouse *Dnmt1 *hypomorph, activation of endogenous retroelements is involved in lymphomagenesis. Presumably, hypomethylation caused by compromise of the DNA methyltransferase leads to unchecked activity of endogenous IAPs which then integrate in the *Notch1 *locus to generate an oncogenic gain-of-function allele [67]. This occurred independently but recurrently in seven of the 16 lymphomas studied.

**Table 1:** Studies describing long interspersed nucleotide element (LINE)-1 hypomethylation in malignant tissues.

Tumor	Evidence for LINE hypomethylation	Reference
Breast cancer	5' flanking sequences of hypomethylated L1 homo sapien elements were isolated from T-47 D cells by inverse polymerase chain reaction (PCR)	[94]

Chronic myeloid leukaemia (blast phase)	Methylation-specific PCR of primary samples; hypomethylation associated with blast crisis, high levels of BCR-ABL messenger RNA, resistance to tyrosine kinase inhibitor chemotherapy	[95]

Chronic lymphocytic leukaemia	A variety of primary specimens analysed by *Hpa*II restriction enzyme digest and Southern blot analysis	[96]

Colorectal adenocarcinoma	As compared to neighbouring normal colon; colorectal carcinomas with sporadic microsatellite instability a noted exception; alternative progression pathways proposed	[96-98]

Hepatocellular carcinoma	Hepatocellular carcinomas compared to surrounding cirrhotic liver; *Hpa*II restriction enzyme digest	[99]

Neuroendocrine tumours	Well-differentiated pancreatic endocrine and carcinoid tumours compared to surrounding tissue; LINE hypomethylation correlates with lymph node metastasis and cytogenetic aberrations	[100]

Prostate cancer	Adenocarcinoma compared to surrounding tissue; hypomethylation of L1 s associated with preoperative PSA, Gleason grade, and clinical stage; associated independently with cytogenetic abnormalities	[101-103]

Urothelial carcinoma	L1 hypomethylation detected by Southern blot in most specimens	[104-106]

While the genotoxic potential of L1 encoded ORF2p has been recognized, a recent paper by Lin *et al*. [68] raised an interesting model suggesting that the protein contributes to tumor development by inducing double-stranded DNA (dsDNA) breaks at specifically targeted sites to which it is recruited. Using chromatin immunoprecipitation in prostate adenocarcinoma cells, the authors demonstrated an androgen ligand-dependent localization of ORF2p to a prostate cancer chromosomal translocation interval. Rather than promoting retrotransposition, their model suggests the endonuclease activity leaves DNA breaks thus subjecting the region to erroneous repair by non-homologous end joining pathways ultimately responsible for the translocation. What factors are responsible for the recruitment and whether ORF2p functions similarly at other breakpoint hot spots in other neoplasias remains unknown.

In addition to the potential role of endogenous TEs in cancer, it should be noted that several laboratories have utilized transposons as tools for cancer gene identification in forward genetic insertional mutagenesis screens in mice. For example, the Sleeping Beauty (SB) DNA transposon system has been successfully used to identify novel cancer genes in tissues that could not be previously analysed by slow transforming retroviruses [69,70]. Recently, this approach has been modified through the conditional activation of the SB in specific tissues [71,72]. With the recent development of a codon-optimized L1 element, it appears that retrotransposons may also serve as useful mutagenesis tools [58,73]. As these elements mobilize by a copy and paste mechanism of retrotransposition, their donor elements are stable. L1 mouse models may also be controlled by tissue-specific promoters and be engineered to contain gene traps [74]. One potential advantage of an unbiased TE-based approach is the ability to study how specific mutations affect tumor cell initiation, progression and maintenance in well-defined, genetically engineered mouse models. Thus, it is likely that these models will provide a complementary approach to cancer genome sequencing studies by uncovering functionally relevant mutations that can further be studied as potential therapeutic targets.

## Strategies for identifying TE insertions

**Figure 1:**
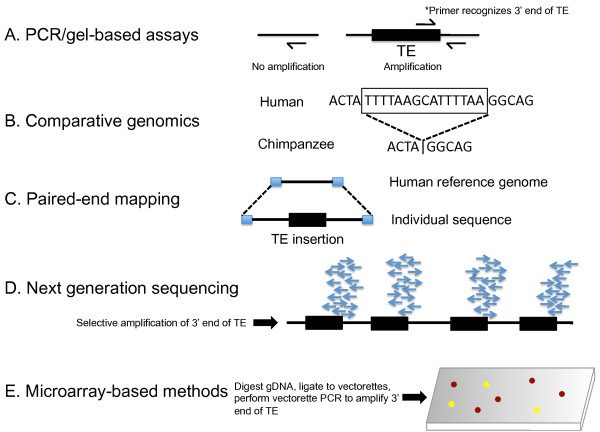
**Techniques for identifying transposon insertions**. (A) Polymerase chain reaction (PCR)-based assays detect transposable element (TE) insertions. L1 display utilizes primers specific to particular subfamilies of LINE-1 elements. Using this method, candidate dimorphic L1 insertions have been identified. The ATLAS technique employs the principles of L1 display and suppression PCR. Genomic DNA is digested and ligated to oligonucleotide primers, and used as a template in a PCR reaction containing L1 and linker-specific primers. Primary PCR products are then used as templates in a linear PCR reaction containing a radiolabelled subfamily-specific L1 primer. Radiolabelled products are detected by electrophoresis and autoradiography. (B) A comparative genomics approach to identify TE insertions and deletions is depicted. For example, the completion of the draft chimpanzee genome sequence provided an opportunity to identify recently mobilized transposons in humans and chimpanzees. If a transposon insertion is present in only one of the two genomes, it is inferred that the insertion occurred since the existence of their most common recent ancestor (~6 million years ago). (C) A paired-end mapping approach is shown. This method entails generating paired-ends of several kilobase fragments, which are sequenced using next generation sequencing methodologies. Differences between the paired-end reads and a reference genomic region reveal the presence of structural variation. Simple insertions and deletions can be detected using this method. (D) A next generation sequencing method is shown. Selective amplification of the 3' end of a transposon is performed followed by deep sequencing. This short-read sequencing approach is able to detect precise insertion positions. (E) Microrray-based methods involve the hybridization of ligation-mediated PCR products to genomic tiling arrays. Specifically, vectorettes are ligated to restriction enzyme-digested genomic DNA. The amplified fragments include the 3' end of a transposon sequence and unique flanking genomic DNA. These amplicons are hybridized to genomic tiling microarrays.

The majority of human genomic transposon sequences are inactive due to the accumulation of mutations and rearrangements that has occurred during evolution, as well as 5' truncations during their insertion that render L1 copies inactive. In the case of the former, these older elements are essentially 'fixed' in human populations today. With all this genomic clutter, identifying polymorphic elements and *de novo *somatic insertions requires directed strategies in order to identify younger, potentially active, transposon copies. Methods for identifying this complement of novel TE insertions have been described and are under rapid development as genomic methodologies continue to avail themselves (Figure 1).

### First generation methods for recovery of novel TEs

Many of the first assays for mobile elements were PCR-based and reliant on gel-based amplicon separation to distinguish the presence or absence of a particular element. Examples include a subtractive suppression PCR assay termed amplification typing of L1 active subfamilies (ATLAS) [75], a random decamer PCR called L1 display [76] and a ligation-mediated PCR called L1 insertion dimorphisms identification by PCR (LIDSIP) [77]. These techniques exploited sequences specific to young L1 families and gave investigators the first insights into the impressive degree of L1 polymorphism in humans. However, they did not lend themselves readily to comprehensive L1 mapping in large numbers of samples.

### Mining genomic sequencing data for TE insertions

Analyses of genomic sequencing data have since contributed significantly to our understanding of polymorphic retroelements in humans, which will presumably accelerate with the ongoing exponential increases in available data. *In silico *mining of the human genome draft [78,79], the Venter genome [80] and comparative sequence analysis of the human and chimpanzee genomes have been performed in order to detect species-specific transposon insertions [81-83]. These studies have revealed that subfamilies of *Alu*, LINE-1 and SVA elements have differentially amplified in humans and chimpanzees. Building on the foundation of the human reference genome, relatively new concerted efforts are underway which may harness sequencing methods to provide insights into structural variation. Paired-end mapping of size selected DNA fragments represents a large-scale approach to identify sizable variants in the genome. For example, using this method with fragments cloned into fosmids, it is possible to detect large insertions and deletions (indels) embedded in repetitive DNA [84,85]. Beck and colleagues recently showed this is a powerful means of identify young, polymorphic full-length L1 s, which are high in retrotransposition activity [86]. Moreover, this method appears to effectively identify the source of parent elements responsible for ongoing L1 insertions in human populations today.

### High throughput TE mapping methods

Technologic developments in sequencing methods and microarray platforms are expanding methods for high throughput TE discovery in the post-genomic era. Several laboratories recently published targeted methods for recovering TE insertion sites that, in combination with high resolution microarrays or deep sequencing, allow researchers to catalogue novel transposition events on a genome-wide basis [63,87-89]. For example, with the Boeke laboratory, we approached L1(Ta) mapping in the human genome using a ligation mediated PCR method known as vectorette PCR [88]. In this method, non-complementary oligonucleotides are ligated to DNA ends and serve to bind a PCR primer only after first strand synthesis is initiated from the L1(Ta). The result is an amplification of unique genomic DNA adjacent to the mobile element. Individual insertion sites can be recognized in this complex mixture of amplicons by labelling and hybridizing to genomic tiling microarrays or by deep sequencing. These data suggest that the rate of new L1 insertions in humans is nearly double previous estimates, with non-parental integrations occurring in nearly 1/100 births, a finding that agrees well with data recently described by Kazazian and colleagues [87]. These types of approaches will undoubtedly be useful in detecting novel TE insertions in both normal individuals and in patients affected with genetic diseases in the future.

## TEs and human genetic variation

To what extent do mobile elements contribute to human genetic diversity? This is a complex question, which is just beginning to be explored in greater depth. Sequencing of the human genome revealed that individual genomes typically exhibit 0.1% variation [2]. Most of the individual genome variation can be attributed to single nucleotide polymorphisms (SNPs), chromosome rearrangements, copy number variants and repetitive elements. The Human Genome Project revealed that there are 2000 polymorphic L1 elements and 7000 polymorphic *Alus *in humans, although it is postulated that the actual number is significantly higher due to ongoing transposition and individual TE polymorphisms. In an effort to detect the degree of genetic variation that is caused by transposable elements, Bennett and colleagues [90] analysed DNA re-sequencing data from 36 people of diverse ancestry. Indel polymorphisms were screened in order to find those that were caused by *de novo *transposon insertions. They estimated that human populations harbour an average estimated 2000 common transposon insertion polymorphisms. In general, these results are consistent with several other studies regarding *Alu *element polymorphisms [8] and L1-Hs insertion polymorphisms [75,76,78,91,92].

In an attempt to identify the number of active polymorphic L1 elements in the human genome, Brouha and colleagues [91] identified 86 young, full-length L1 elements from an early draft of the human genome sequence. Of these, they determined that 38 (44%) are polymorphic for presence in the human genome. In addition, a similar number of elements were identified to be active in a cell-culture based retrotransposition assay. Based on these results, it is estimated that there are 80-100 active L1 s in the average diploid genome. Of these, *in vitro *retrotransposition assays suggest only a small number are highly active and have accounted for the majority of *de novo *insertions [91].

Recently, several groups have focused their efforts on determining what fraction of structural variants (SVs) in the human genome is due to TE sequences. Korbel and colleagues [84] employed a paired-end mapping technique to identify ~1000 SVs and reported that the number of these variants in humans is significantly higher that originally appreciated. Xing *et al*. [80] analysed ~8000 SVs with the goal of identifying those that are associated with mobile elements. Computational analyses and experimental validation revealed that roughly 700 novel transposable element insertion events due to *Alus*, L1 elements and SVAs are found in an individual diploid genome. Transposon-mediated deletions were also detected. The Jorde laboratory recently demonstrated that the presence of a fixed *Alu *insertion is predictive of an elevated local recombination rate, which may further contribute to non-allelic recombination events [93]. Indeed, it has become increasingly apparent that TEs play an important role in the generation of structural variants between individuals and this is an exciting area ripe for further study. Future efforts focused on characterizing the full extent of mobile element associated structural variants and probing their potential functional consequences are warranted.

## Conclusions

Our understanding of the basic biology of TEs has expanded dramatically in the 60 years since their initial discovery. Yet, there are still many open questions awaiting further study. For example, the mechanisms of transposon regulation and mobilization in the germline and somatic cells have not been fully elucidated. If we appreciate where, when and how these processes occur, we will ultimately better understand the impact of these elements on host genomes and the extent to which they contribute to diversity.

Although major advances have been made in the identification of transposon insertions in humans, we are at the very earliest stages of recognizing the full implications of these findings. It is clear that TE insertions provide a rich source of inter-individual genetic variation. With the continued optimization of technologies that are able to identify all transposon insertions, we will undoubtedly gain a better understanding of the extent of TE diversity in individual genomes, in human populations and in disease states.

## Abbreviations

APOBEC: apolipoprotein B messenger RNA editing enzyme; Catalytic polypeptide; ATLAS: amplification typing of L1 active subfamilies; IAP: intracisternal A particle; indels: insertions and deletions; LINE: long interspersed nucleotide element; LTR: long terminal repeat; MAEL: MAELSTROM; PCR: polymerase chain reaction; PIRNA: piwi-interacting RNA; SB DNA: Sleeping Beauty DNA; SINE: short interspersed nucleotide element; SV: structured variant; TE: transposable element.

## Competing interests

The authors declare that they have no competing interests.

## Authors' contributions

KAO and KHB wrote the review. Both authors approved the final manuscript.
